# Perceptions and attitudes of emergency department nurses toward artificial intelligence applications in triage: a qualitative study

**DOI:** 10.3389/fpubh.2026.1795233

**Published:** 2026-03-16

**Authors:** Xinrui Mao, Na Liu, Hang Wang, Qi Wang, Shuqin Xia, Xiaoya Ma, Lu Yin

**Affiliations:** 1 Emergency Department, Shanghai East Hospital, Shanghai, China; 2 School of Medicine, Tongji University, Shanghai, China; 3 Shanghai East Hospital, School of Medicine, Tongji University, Shanghai, China; 4 School of Nursing, Hubei University of Medicine, Shiyan, China

**Keywords:** artificial intelligence, emergency department nurses, nurse, perceptions, qualitative study

## Abstract

**Object:**

This study aims to explore the cognition and attitude of emergency department nurses toward the application of artificial intelligence (AI) in triage, reveal the challenges faced in the application process, and provide suggestions for promoting the application of AI triage in China.

**Methods:**

This study adopted a qualitative research design and employed the Colaizzi phenomenological method. Purposive sampling was used to select emergency department nurses from September 2025 to December 2025 for semi-structured in-depth interviews.

**Result:**

A total of 18 research subjects were included in this study, 2 themes and 6 sub-themes were identified: (1) Nurses’ cognition of the application of AI triage, including reducing work pressure, having concerns, and the boundary uncertainty of human-AI collaboration; (2) Nurses’ demands for the application of AI in triage include data security and information accuracy, clinical adaptation and data intercommunication, as well as ethical guarantees.

**Conclusion:**

Nurses have a relatively rational understanding of the application of AI in triage, which can bring positive impacts to the triage process, but they also face multiple challenges. The development and application of future AI triage systems should focus on the demands of patient safety, data security, and information accuracy, to enhance the efficiency of emergency triage and alleviate the pressure on nurses.

## 1 Introduction

The emergency department serves as the core hub for receiving critically ill patients and constitutes a vital component of the healthcare delivery system. Its transport efficiency directly impacts patient safety and service quality (1). Currently, emergency department overcrowding represents a significant challenge in global healthcare (2). Relevant studies indicate (3) that emergency departments universally face rising patient volumes, with annual growth rates reaching 8 to 12%, resulting in delayed treatment for some critically ill patients and missed opportunities for optimal care. In China, emergency departments face increasing pressure due to the large population base and accelerating aging. Triage, as the primary step in the emergency care process, is responsible for rapidly assessing conditions, prioritizing patients, and efficiently allocating resources (4). Triage nurses must synthesize multidimensional information—including patient complaints, vital signs, and medical history—within 3 to 5 min to accurately complete condition triage. However, factors such as work pressure and varying levels of experience can lead to fatigue among triage nurses, resulting in decreased efficiency. Multidisciplinary teams (MDT) (5), five-tier triage systems (6), and the Emergency Severity Index (ESI) (7) are commonly employed domestically and internationally to enhance triage efficiency and accuracy. However, these approaches still rely heavily on triage nurses for implementation and persistently face challenges such as excessive triage duration, making them ill-suited to meet the demands of high-quality emergency care development. With the rapid advancement of AI technology in the medical field, AI-assisted triage systems can integrate multi-source medical data. Through machine learning algorithms trained on vast amounts of emergency case data, they can swiftly identify high-risk patient characteristics and automatically match triage criteria (8). Various models of intelligent triage systems have been developed both domestically and internationally, including clinical decision-based triage systems (9), deep learning-based triage systems (10), and traditional scale-based triage systems (11). In China, due to factors such as resource distribution, clinical adaptability, and cognitive differences, most studies remain confined to laboratory validation and small-scale pilot phases. Triage models continue to be primarily nurse-led, with only a small number of hospitals implementing intelligent triage systems. Nurses lack systematic training in AI medical technology, and their cognitive attitudes cannot directly refer to international research conclusions. As the primary executors of triage and core users of AI triage systems, triage nurses’ perceptions and attitudes toward intelligent triage models provide valuable insights into both barriers and enablers for system adoption. At present, some scholars have explored the attitudes and perceptions of triage nurses toward the AI triage system under different medical systems. However, most studies merely describe the attitude tendencies of nurses, without delving into the underlying influencing factors behind their cognitive attitudes, nor systematically sorting out the core demands of nurses for the AI triage system. Therefore, this study aims to explore emergency triage nurses’ perceptions and attitudes toward AI applications in triage within the Chinese cultural context, thereby providing evidence for the clinical translation of AI technology in triage settings.

## 2 Methods

### 2.1 Study design and participants

This study employed a phenomenological qualitative research design, utilizing semi-structured in-depth interviews to explore emergency department nurses’ perceptions and attitudes toward the application of AI in triage. The aim is to explore the experiences of emergency department nurses regarding the application of AI in triage and to uncover the reasons behind their attitudes. Conducted from September to December 2025 at two tertiary hospitals in Shanghai, purposeful sampling was used to select participants from nurses responsible for triage in emergency departments, to enhance the depth, relevance and information saturation of the research findings. The entire research process strictly adhered to the Core Quality Reporting Standards for Qualitative Research (COREQ) (12) to ensure the standardization and reproducibility of the research methodology.

#### 2.1.1 Inclusion and exclusion criteria

Inclusion criteria: (1) Currently employed registered nurses engaged in emergency triage duties. (2) At least 3 months of triage experience in the emergency department. (3) Prior use of or familiarity with intelligent triage systems during work. (4) Informed consent and voluntary participation in this study. Exclusion criteria: (1) Trainee nurses, interns, or rotating nurses. (2) Nurses currently engaged in nursing management, teaching, or research in the emergency department, not frontline clinical work; (3) Nurses with less than 3 months of experience in emergency triage; (4) Nurses unable to fully participate in the interview or express their views clearly due to physical, psychological, or work-related reasons; (5) Nurses directly involved in the design or promotion of the intelligent triage system.

#### 2.1.2 Determination of sample size

Sample saturation was determined based on the principle of data saturation, specifically when no new themes emerged across three consecutive interviews (13). A total of 24 nurses were invited to participate in this study. Among the 24 invited nurses, 20 agreed to participate and underwent qualitative interviews. Two conducted pre-interviews; since pre-interviews primarily aim to test the scientific validity, rationality, and feasibility of the interview guide (14) rather than collect formal data, their interview content was not included in the final research findings. Starting with the 16th nurse interviewed, no new themes emerged in the results of three consecutive interviews. Therefore, 18 nurses were ultimately included.

### 2.2 Data collection method

First, the research team collaborated with head nurses from the emergency departments of two hospitals to recruit participants. With their assistance, based on the actual job positions of the on-duty triage nurses in the department, their years of experience in emergency triage, and their experience in using the AI triage system and other basic information, potential participants who met the standards were initially screened out without involving personal privacy, and an anonymous candidate list was provided to the research team. Based on the anonymized candidate list, researchers who have received qualitative research training will conduct regular departmental meetings and issue formal research recruitment announcements. At the same time, they will reserve their work contact information for potential participants to consult. If potential participants are willing to participate, they can proactively contact the researcher. The researcher will conduct a second qualification check on them, confirming one by one whether they fully meet the inclusion and exclusion criteria. Those who pass the check will be included as candidate interviewees. Next, the purpose and significance of this study were explained to them, and their willingness to participate was sought. Before the formal interview, the participants signed the informed consent form. We collected general sociodemographic information, including gender, age, educational attainment, professional title, years of service, and previous experience in using the intelligent triage system. This data was crucial for analyzing emergency department nurses’ perceptions and attitudes toward AI applications in triage. Finally, data was gathered through face-to-face, semi-structured in-depth interviews conducted in quiet conference rooms. The entire interview was conducted in Chinese, which was in line with the daily working communication language of the research subjects, ensuring that the participants could freely and accurately express their own cognition and experiences. Each interview was conducted by two researchers, all of whom had received training in qualitative research theory and possessed the necessary skills to conduct such studies. Interviews lasted between 24 and 52 min. With participant consent, all interviews were audio-recorded. Researchers also observed and documented participants’ verbal expressions and nonverbal cues. In response to the situation where participants’ expressions were ambiguous and their emotional expressions were implicit, an open exploratory questioning approach was adopted to explore their deep emotional experiences regarding AI triage, ensuring the authenticity and depth of the information. The interview content is transcribed within 24 h. After removing all personally identifiable information, the complete transcript of the interview is returned to the corresponding participants online. The researchers invited the participants to carefully check the transcribed content, with a focus on confirming whether the interview records were complete, whether the semantics were accurate, and whether there were any transcription deviations caused by slips of the tongue or unclear recording. They also allowed the participants to correct, supplement or delete the content. After receiving feedback from the participants, the research team conducted the final calibration of the transcript and analyzed the data. The data reaches saturation when no new topics are extracted in three consecutive interviews.

### 2.3 Interview guide

According to the research purpose, a semi-structured interview outline was compiled, and five emergency department experts were consulted, including two associate chief physicians of emergency medicine and three head nurses of the emergency department. Ask whether the interview outline comprehensively covers the research scope. Is there any information that deviates from the research topic? Is the expression of the question accurate? Subsequently, five experts independently put forward suggestions for revision. The research team held an online meeting to discuss different opinions together. The initial draft of the interview outline was revised, including optimization of question expression, adjustment of logical sequence, and supplementation of clinical scenarios. Then, pre-interviews were conducted with the two recruited participants, and adjustments were made based on their feedback to ensure clarity and practicality. The core issues of the final interview outline are in Table 1.

**Table 1:** Interview guide.

Interview questions
Basic cognition
(1) How well do you know about the AI triage system? What are the main channels to learn about this system?
(2) What do you think should be the core function of an AI triage system? What is the most prominent difference compared with traditional manual triage?
Those with usage experience
(3) How do you think the system performs in terms of operability, accuracy of disease classification, and compatibility with emergency clinical needs? In which scenarios does it perform well/poorly? Could you give some examples to illustrate?
(4) What advantages and limitations do you think the AI triage system has in emergency work?
Those without usage experience
(5) Have you ever taken the initiative to learn about the AI triage system? What are your understandings of its core working principle and application effects?
(6) If your hospital introduces an AI triage system, would you be willing to give it a try? What are the core expectations and concerns respectively?
Attitude and demand
(7) What’s your attitude toward AI-assisted triage? Can this model improve the efficiency and accuracy of triage?
(8) What do you think is the most crucial condition for the application of an AI triage system?
(9) What do you think are the prerequisite conditions for promoting an AI triage system?
Others
Do you have any other information that needs to be supplemented?

### 2.4 Research team

The research team is composed of experienced nursing experts and postgraduate nursing students, including 4 intermediate researchers and 3 postgraduate students. The first author and the second author were mainly responsible for the research design, interviews and data analysis. During the interview, one of the two researchers was responsible for the interview, while the other assisted in observing and recording. The two researchers who conducted the interviews were both non-clinical administrative staff and had no superior-subordinate relationship with the interviewed nurses. All researchers jointly participated in the discussion of dissenting opinions. When consensus was not reached, the decision was made by the corresponding author, Lu Yin, who is an expert in the field of emergency medicine and has professional knowledge in this field to ensure the scientific and accuracy of data analysis. All members of the team have received qualitative research training and possess rich clinical experience, ensuring the rigor and scientific effectiveness of the research.

### 2.5 Data analysis method

The transcribed text was imported into the NVivo 15.0 software for analysis. The data analysis followed the Colaizzi seven-step analysis method. The specific steps included familiarizing with the text, identifying meaningful statements, constructing code, clustering topics, providing detailed descriptions, generating basic structures and verifying structures (15). The researchers read the transcribed texts many times to familiarize themselves with the data. To enhance the credibility of the process, two researchers who had received qualitative research training independently carried out the initial coding. When the two researchers had differences during the result coding process, these issues were resolved through repeated discussions among the seven researchers until a consensus was reached on the final topic. Finally, to verify whether the topic could reflect the true viewpoints of the participants. We began the verification within 1 week after the data collection was completed to ensure that the research subjects had a clear memory of the interview content. We informed the original participants of the formed themes and asked them whether these themes were in line with their understanding and feelings about the application of AI triage. Whether the original interview data selected for each topic is accurate and whether there are any key cognitions, attitudes and needs that have not been mentioned. All the participants believed that these topics were in line with their viewpoints. In addition, the entire research team discussed the entire analysis process and ensured that the final topic clearly reflected the data from the interviews.

### 2.6 Rigor

This study followed the guidelines for qualitative research reports (12). A multi-dimensional research rigor guarantee system has been constructed based on the four major standards of credibility, dependence, confirmability and transferability.

#### 2.6.1 Credibility

This study adopted a semi-structured in-depth interview approach to collect data, delving into core issues layer by layer to ensure that the obtained information closely aligns with their real clinical experiences. Dual verification is implemented simultaneously in transcription and topic extraction. In addition, reflective research notes are recorded. After each interview, the two researchers who conducted the interview jointly write notes, documenting potential researcher biases during the interview process, non-verbal cues from the research subjects, etc. Regular discussions are held within the team to optimize subsequent interview strategies.

#### 2.6.2 Dependability

This study optimized the research tool through pre-interviews and revised the interview outline based on the feedback. At the same time, standardized data analysis methods are adopted, and the entire process strictly follows the Colaizzi seven-step analysis method for data processing.

#### 2.6.3 Confirmability

During the interview, only neutral follow-up questions will be conducted, and no guiding or augmentative remarks will be made. At the same time, all research materials such as anonymized interview recordings, transcriptions, encoded records, expert consultation opinions, and reflective notes should be uniformly archived.

#### 2.6.4 Transferability

The research subjects included in this study were nurses who used AI triage and those who did not, presenting the demographic characteristics of the research subjects in full. In addition, for each topic and sub-topic, corresponding interview quotes from the research subjects are provided to visually present the data sources of the results, offering a reference basis for other related studies with similar medical backgrounds.

### 2.7 Ethical considerations

This study has been approved by the Ethics Committee of Shanghai East Hospital Affiliated to Tongji University (2025-095). Throughout the entire research process, all research subjects were informed in detail of the research purpose, interview content, data usage and privacy protection measures, and the research subjects signed the written informed consent form in person. At the same time, it is clearly informed to the research subjects that they have the right to withdraw from the research unconditionally and independently throughout the process. The withdrawal application does not need to provide any reasons. After withdrawal, their relevant research data will be immediately destroyed and will not have any impact on their professional or clinical work. All audio recordings and written records are for research and analysis purposes only and may not be used for any other purposes without the permission of the participants.

## 3 Result

### 3.1 Demographic characteristics of the participants

A total of 18 eligible emergency department nurses participated in this study (Table 2). Among them, there are 2 males and 16 females. The participants’ ages ranged from 24 to 48 years old, with an average age of 33.67 years old. The working years in the emergency department range from 1 to 17 years, with an average of 7.67 years. In terms of educational attainment, 14 people have a bachelor’s degree or above, accounting for 77.78%. In addition, 55.56% of the participants have used the intelligent triage system.

**Table 2:** General information of respondents.

Variable		Frequency (*n*)	Percentage (%)
Age (year)	<30	6	33.33
	30~40	8	44.44
	>40	4	22.22
Gender	Man	2	11.11
	Woman	16	88.89
Educational level	Junior college	4	22.22
	Undergraduate	13	72.22
	Master’s degree	1	5.56
Professional title	Junior	12	66.67
	Intermediate	6	33.33
Emergency working hours	<3 years	3	16.67
	3~5 years	5	27.78
	6~10 years	3	16.67
	>10 years	7	38.89
Have you ever used intelligent triage?	Yes	10	55.56
	No	8	44.44

### 3.2 Theme 1: nurses’ perspective on the application of AI in triage

#### 3.2.1 The application of AI in triage can relieve work pressure

Nurses believe that the application of AI technology in the field of emergency triage can enhance the efficiency of triage and reduce work pressure. Especially in the context of China, with the intensification of population aging and the increase in the number of patients seeking medical treatment, the application of AI in triage can shorten the decision-making time for nurses and alleviate their work pressure. In addition, nurses who have used the AI triage system believe that the intelligent triage system, after standardized assessment, has reduced the differences in experience during manual triage and the subjective biases caused by fatigue.

*N3: It can automatically match the classification criteria based on the basic information of patients and provide suggestions quickly, shortening the time for us to make decisions.*

*N8: These systems must all be classified based on guidelines and scales. No matter who uses them or when they are used, they can all be classified according to the pre-designed standards.*

These positive perceptions represent nurses’ recognition of the core value of AI in the triage field, enabling them to focus on more professional tasks such as complex case assessment and humanistic care.

#### 3.2.2 Nurses have concerns about the application of AI in triage

Nurses have multiple concerns about the application of AI in triage, which mainly focus on patient safety, data security, and the actual clinical operation effect. There is concern that the accuracy of AI in classifying complex cases may be difficult to guarantee, which could lead to potential safety hazards for patients. Meanwhile, AI triage needs to record patients’ personal information. Nurses are concerned that system storage vulnerabilities may lead to the risk of information leakage, which violates the principle of medical privacy protection. In addition, in China, due to reasons such as uneven distribution of medical resources, medical institutions generally conduct triage manually. Most nurses have not used the intelligent triage system, so they have raised concerns about the practicality of the system, worrying that the operation of the system is not smooth enough and the operation page is too complicated, which has intensified the pressure of triage.

*N17: It’s not that we do not want to trust AI; it’s mainly that we do not know what its judgment logic is. Only by clarifying these can we use it with confidence.*

*N15: AI records sensitive information such as patients’ names, ages, and past medical histories, all of which involve privacy. There is a concern that this information might be leaked, causing major problems.*

*N1: I am afraid that during peak hours, patients will queue up and the system will lag, and the entered information will not be saved. Then we must do manual triage, which increases our workload.*

These concerns stem from the obstacles to the implementation of the AI triage model. The fast pace of the emergency department places high demands on the convenience and stability of the system.

#### 3.2.3 Nurses have uncertainty about the human-AI collaboration boundaries

In China, influenced by the traditional triage model, AI triage systems have not been widely applied in medical institutions in our country, and the triage model is mainly led by nurses. The interviewed nurses have uncertainties about the boundaries of human-AI collaboration in the application of AI triage. Specifically, they lack relevant training, have unclear definitions of specific task scopes, unclear divisions of human-AI decision-making rights, and unclear attribution of legal responsibilities during collaboration. From the perspective of sociotechnical systems theory and human factors engineering, this kind of uncertainty is a typical cognitive dilemma in the integration of medical technology and clinical practice. Nurses who do not use AI for triage are not clear whether they should be in the leading decision-making or auxiliary verification position during the triage process, nor are they sure about the specific triage tasks they need to undertake in the human-AI collaboration mode. Nurses who have used the AI triage system show that during the usage process, when the triage results of the system are inconsistent with their own clinical experience judgments, they have the authority to make independent adjustments, but there is still no clear norm for the boundary of this adjustment right. Furthermore, throughout the entire human-AI collaboration process, there is a lack of clear boundaries of responsibilities and unified operational norms. Once medical issues arise, the responsible entity between humans and AI is not clear.

*N7: Our hospital does not have any relevant training accompaniment yet. If the hospital uses it, we have no idea how to use it.*

*N6: The current system is still not perfect. I’m not sure what tasks I’m mainly responsible for in this triage process. Should I take the lead or should AI take the lead?*

Nurses’ vague understanding of the application of AI triage is the current predicament faced by the application of AI in emergency triage and the key reason for various concerns. The AI triage mode has broken the original working mode and formed a new system of human-machine collaboration. However, it has not yet established clear boundaries of human-machine roles and standards for the division of collaboration rights and responsibilities, which has further intensified concerns about the safety and practicality of collaboration.

### 3.3 Theme 2: nurses’ demand for the application of AI in triage

#### 3.3.1 Nurses’ demands for data security and information accuracy

Data security is an unbreakable bottom line. Due to nurses have concerns about data security and information accuracy, they show certain demands for data security and information accuracy. In clinical work, it is clearly required to protect patients’ privacy. As AI technology stores patient information through systems, there may be a risk of data leakage. Once data is leaked, patients’ daily lives may be affected and even cause psychological concerns. Therefore, nurses hope that the application of AI can also ensure the security of data. Some nurses found that the AI results did not match the actual conditions of the patients when using AI for triage. They hope that AI can accurately identify the patients’ chief complaints and reduce the triage deviation caused by inaccurate information collection.

*N13: Computers are often shared by multiple people. Occasionally, there may be situations such as forgetting to lock the screen, which could lead to information leakage. It is hoped that it can automatically issue a warning when suspicious data export behavior is detected.*

*N10: If AI can proactively generate more specific questions to guide patients based on the provided information, precise triage will be possible.*

It can be seen from these that nurses’ demands for data security and information accuracy are the fundamental demands raised by security issues. Accurate information is the core capability requirement for AI to undertake information processing work in triage. Through technical means, data leakage early warning and privacy protection are achieved, laying a foundation for the subsequent development of collaboration.

#### 3.3.2 Nurses’ demands for clinical adaptability and data intercommunication

Nurses have certain concerns about the applicability of AI in triage, so nurses have a great demand for the clinical adaptability of AI application in triage, and these demands mainly focus on the convenience of functional operation and the applicability of assessment methods. Nurses generally believe that emergency departments are characterized by a fast pace, complex patient conditions and many unexpected situations. Systematic assessment needs to consider the influence of various clinical factors. Meanwhile, it is hoped that the operation functions are convenient to improve the accuracy of triage for patients during peak hours. In addition, some nurses who have used AI triage hope that the data can be shared with the hospital’s medical equipment and information system data, which can reduce the nurses’ work pressure.

*N18: Some of the patients here have rather complex conditions. Through our manual assessment in multiple aspects, if we were to switch to an AI triage system, this system would need to have the ability to evaluate* var*ious diseases.*

*N5: Once, when I was inputting information for a patient, many Windows popped up one after another. When it was completed, there were already five or six patients queuing up behind.*

*N9: If the data can be integrated with the hospital system, after the basic chief complaint of the patient is entered into the system, there is no need to re-fill it in the subsequent diagnosis and treatment process, which reduces the work pressure.*

These demands mainly stem from the practical adaptation obstacles and insufficient collaboration in the process of AI application. Clinical adaptation requires that the AI system be in line with the characteristics of the emergency department to achieve convenient operation. Data intercommunication needs to be adapted to the macro collaboration of the hospital’s overall medical system to truly enhance the efficiency of collaboration.

#### 3.3.3 Nurses’ demand for ethical safeguards

Ethical assurance is the foundation that runs through the entire medical process and is of vital importance in the context of AI applications. When the suggestions of AI deviate from the clinical experience of nurses, there need to be definite standards to safeguard the medical rights and interests of patients. Furthermore, as a computer system, AI lacks overall consideration of patients’ emotions, social backgrounds, etc. during the triage process of patients. Therefore, triage nurses need to be present to provide patients with humanistic care within their capacity.

*N7: What if the triage is carried out strictly according to the system prompts and the condition is delayed? Who is the subject of this responsibility? There need to be clear norms.*

*N16: Besides listening to patients’ chief complaints and triaging them, our job also involves observing their emotional states and providing them with assistance within our capacity. All these are beyond the reach of AI.*

These demands aim to alleviate nurses’ various collaboration concerns by establishing clear institutional norms to define the role boundaries, decision-making priorities, and responsibility attribution in AI triage human-machine collaboration.

## 4 Discussion

Nurses’ understanding of the application of AI in triage is characterized by a rational perception of its core value and potential risks, but they show a vague understanding of their own responsibilities under the AI triage model. In China, the annual growth rate remains at 8% to 12% (16). Triage nurses need to complete the integration of information and classification of patients’ conditions within 3 to 5 min. Meanwhile, there are differences in medical standards among various regions (17), and there are also differences in the levels of tertiary hospitals and primary medical institutions (18). In this study, nurses were able to recognize that AI plays a positive role in triage. The AI triage system can integrate patient information, quickly match and grade, effectively relieve the work pressure on nurses during peak hours, and at the same time reduce triage deviations caused by differences in nurses’ experience. Wang et al. (19) developed an intelligent triage system by using the large language model ChatGPT, and evaluated the accuracy, consistency and feasibility of patient triage using the MEWS score. It was found that the large language model showed high accuracy in guiding patient triage. However, some nurses who had used AI triage in this study also raised some concerns. In clinical practice, emergency departments often deal with patients with complex conditions. Such patients usually need to be comprehensively judged based on clinical experience and dynamic vital signs. However, the AI triage model is based on pre-set algorithms and large model data for standardized assessment, which may deviate from the actual condition of the patients, thereby leading to inaccurate results. This is consistent with the results of the study by Tahernejad et al. (20). This study points out that AI is prone to false negative results in the judgment of complex cases in emergency triage, especially underestimating the severity classification of older adults patients with comorbidities, which increases the risk of clinical missed diagnosis and adverse medical events. In addition, during the application of AI, it is necessary to collect and store sensitive information such as patients’ names, medical histories, chief complaints, and contact details. As a system and algorithm, AI may have the risk of stored information leakage at any time. Therefore, data security is of great significance to both patients and medical institutions. It can be seen from this that nurses have a rational understanding of the application of AI in triage. Before applying AI in the field of triage, it is necessary to establish a model database with sufficient sample size and conduct strict tests to ensure the accuracy of triage. At the same time, a complete data security storage system should be established, and a series of management permissions should be set to reduce the risk of information leakage.

The cognitive ambiguity problem existing among nurses in the application of AI triage is the result of the conflict between the traditional manual triage mode and the new human-machine collaboration mode. The main manifestations are unclear self-positioning and ambiguous professional boundaries. These problems not only affect the application effect of AI in triage but also the quality of emergency medical care (21). In China, nurses are the leaders in disease assessment and classification decision-making. They make judgments based on their own medical knowledge and clinical experience. This role has formed a stable working mode in long-term clinical practice (22). The application of the AI triage model can replace nurses in completing data integration, disease analysis, and preliminary classification work. However, this model conflicts with the role that nurses play in triage. Nurses who have not used AI triage are confused about the work content they need to undertake and are not clear about what they are responsible for. At the same time, when the conclusions of AI conflict with those of nurses, should nurses take the lead or should AI take the lead? Mani and Albagawi (23) reviewed the literature on the application of AI in emergency care and found that AI has potential in triage, but there are also algorithmic biases and challenges. Nurses need to conduct critical evaluations when adopting AI suggestions. Qiu et al. (24) also concluded through an observational study that the accuracy of AI in triage is average and it tends to over-triage. Currently, the professional judgment of nurses (especially for critically ill patients) cannot be completely replaced. It can be seen from this that nurses still play an irreplaceable role in the AI triage process. Nurses not only need to guide patients to complete the input of AI data but also need to judge the decisions generated by AI to ensure the accuracy of triage. In addition, nurses who have used AI triage are more likely to have a vague understanding of professional boundaries, which is also a reason for their unclear understanding of their own roles. When AI triage is applied, there are problems such as unclear responsibility attribution and ambiguous decision-making authority. When junior nurses encounter patients with complex conditions, due to their lack of experience, they may tend to follow the results of AI. Although senior nurses have strong judgment abilities, they hesitate due to the lack of clear norms to support them, fearing that disputes might arise after adjusting the results. This not only reduces the efficiency of triage but also poses potential safety hazards. Therefore, before AI is applied in the field of triage, medical institutions need to formulate clear operational norms to safeguard the rights and interests of nurses and patients.

The multi-dimensional core demands raised by nurses in response to the application obstacles of AI triage have pointed out specific practical directions for the localization optimization and promotion of the AI triage system. It is necessary to build a clinical transformation support system for AI triage from dimensions such as technical optimization, ethical governance, training of nursing staff, and clarification of professional roles. Hassanein et al. (25) found that AI can help nurses reduce their burden, but it still needs improvement in aspects such as data privacy, algorithms, and clinical judgment. In this study, nurses who have used AI for triage have a significant demand for the accuracy of information. When facing patients with complex conditions, AI cannot merely collect fixed information for judgment. It is necessary to optimize the information collection logic of the AI system, develop an intelligent guiding questioning function, and automatically generate specific questions for vague patient complaints to help patients clearly express their conditions. Reduce information loss. In addition, nurses also have a strong demand for data security and clinical adaptability. In the development and application of AI systems, the focus should be on nurses and patients, reducing the trivial processes of the system. Technically, measures such as encrypted storage, operation traceability, and suspicious behavior early warning should be adopted to lower the risk of data leakage. At the ethical governance level, it is necessary to build an AI triage ethical governance system that conforms to the Chinese medical system based on nurses’ ethical guarantee demands for responsibility division and medical norms and clearly define the technical application boundaries of AI triage and the responsibility division standards for human-machine collaboration. At the level of clarifying professional roles, it is necessary to define the core decision-making subject status and specific job boundaries of emergency nurses in the human-machine collaboration of AI triage through institutional forms, fundamentally solving the predicament of nurses’ ambiguous role cognition. At the level of nursing staff training, it is necessary to carry out systematic and hierarchical AI triage specialized training considering the current situation where nurses lack knowledge of AI medical technology, to enhance nurses’ AI application ability and human-machine collaboration quality and fill the gap in the existing training system.

## 5 Limitations

This research is a qualitative one and has some limitations that need to be improved in future studies. Firstly, the samples of this study were from two tertiary hospitals in Shanghai, one of which has already adopted an intelligent triage system. The nurses participating in the research may be potentially influenced by the development of the institution, resulting in social expectation bias and a tendency to express positive evaluations in the interviews. Meanwhile, the regions where they are located are mainly economically developed areas. Nurses can meet digital medical tools such as intelligent nursing terminals in their daily work and have a certain foundation in the use of digital medical care. Their acceptance and awareness of AI technology are much higher than those of medical staff in low-resource areas. Therefore, the proportion of nurses who used the intelligent triage system in this study was relatively high, which might affect the promotion of the research results in other environments. Secondly, this study is a qualitative one. Data was collected through semi-structured interviews, and subjective factors may have influenced the process of data collection and processing. Thirdly, there may be an interviewer effect. Researchers’ own prior cognitive tendencies toward AI triage may unintentionally generate subtle suggestive expressions during communication. Additionally, in this study, the recruitment of research subjects was completed with the assistance of head nurses, which might cause participants to have psychological concerns during the interview process. Fourth, the research subjects of this study are from China. The interviews were conducted in Mandarin, while the data analysis and article writing were carried out in English, which may lead to bias. Finally, the perspective of this study focused on nurses, and the sample size was small, not including patients, doctors, and hospital administrators, which might lead to incomplete research results. Therefore, in the future, the sample size in different regions should be expanded to conduct multi-perspective views on the application of AI in triage for patients, doctors, hospital administrators.

## 6 Conclusion

This study explores the cognitive attitudes and demands of emergency nurses toward the application of AI triage in the context of Chinese medical culture. The fundamental reasons for nurses’ vague understanding of AI in triage have been clarified. By analyzing the perception of nurses with experience in using AI triage systems and those without, the challenges of implementing AI triage systems have been revealed, providing more comprehensive and systematic empirical evidence for the high-quality and sustainable clinical transformation of AI triage technology in China.

## Data Availability

The original contributions presented in the study are included in the article/Supplementary material, further inquiries can be directed to the corresponding author.

## Supplementary material

Supplementary Table S1 General information of respondents.
