# Supplementary material for: Perceptions and attitudes of emergency department nurses toward artificial intelligence applications in triage: a qualitative study
Source: Front Public Health. 2026 Mar 16;14:1795233. doi: 10.3389/fpubh.2026.1795233 (PMC13033648; doi:10.3389/fpubh.2026.1795233)
Supplement: Supplementary Table S1 — General information of respondents. [file Table_1.docx]

**Table 1 General information of respondents**

| Number | Gender | Age | Education level | Professional title | Emergency working hours | Have you ever used the intelligent triage system？ |
| --- | --- | --- | --- | --- | --- | --- |
| N1 | Female | 29 | undergraduate | Junior | 4 | Yes |
| N2 | Female | 36 | undergraduate | Intermediate | 11 | No |
| N3 | Female | 24 | Junior college | Junior | 1 | Yes |
| N4 | Female | 41 | undergraduate | Intermediate | 12 | Yes |
| N5 | Male | 33 | undergraduate | Junior | 6 | No |
| N6 | Female | 28 | undergraduate | Junior | 3 | No |
| N7 | Female | 45 | undergraduate | Intermediate | 14 | No |
| N8 | Female | 31 | Master's degree | Intermediate | 4 | Yes |
| N9 | Female | 27 | undergraduate | Junior | 3 | No |
| N10 | Female | 39 | Junior college | Junior | 13 | Yes |
| N11 | Female | 25 | Junior college | Junior | 2 | No |
| N12 | Female | 34 | undergraduate | Junior | 8 | Yes |
| N13 | Male | 30 | undergraduate | Junior | 5 | No |
| N14 | Female | 48 | undergraduate | Intermediate | 17 | Yes |
| N15 | Female | 32 | undergraduate | Junior | 7 | Yes |
| N16 | Female | 37 | Junior college | Junior | 12 | Yes |
| N17 | Female | 24 | undergraduate | Junior | 1 | No |
| N18 | Female | 43 | undergraduate | Intermediate | 15 | Yes |
